# Probiotics combined with rifaximin influence the neurometabolic changes in a rat model of type C HE

**DOI:** 10.1038/s41598-021-97018-8

**Published:** 2021-09-09

**Authors:** Emmanuelle Flatt, Valérie A. McLin, Olivier Braissant, Katarzyna Pierzchala, Paola Mastromarino, Stefanita-Octavian Mitrea, Dario Sessa, Rolf Gruetter, Cristina Cudalbu

**Affiliations:** 1 Laboratory for Functional and Metabolic Imaging, Ecole Polytechnique Fédérale de Lausanne (EPFL), Lausanne, Switzerland; 2 Swiss Pediatric Liver Center, Department of Pediatrics, Gynecology and Obstetrics, University Hospitals Geneva, University of Geneva, Geneva, Switzerland; 3 Service of Clinical Chemistry, Lausanne University Hospital, University of Lausanne, Lausanne, Switzerland; 4 CIBM Center for Biomedical Imaging, Lausanne, Switzerland; 5 Animal Imaging and Technology, Ecole Polytechnique Fédérale de Lausanne (EPFL), Lausanne, Switzerland; 6 Section of Microbiology, Department of Public Health and Infectious Diseases, Sapienza University of Rome, Rome, Italy

**Keywords:** Experimental models of disease, Liver diseases, NMR spectroscopy

## Abstract

Type C hepatic encephalopathy (HE) is a neuropsychiatric disease caused by chronic liver disease. Management of type C HE remains an important challenge because treatment options are limited. Both the antibiotic rifaximin and probiotics have been reported to reduce the symptoms of HE, but longitudinal studies assessing their effects on brain metabolism are lacking and the molecular mechanisms underpinning their effects are not fully understood. Therefore, we evaluated in detail the effects of these different treatments on the neurometabolic changes associated with type C HE using a multimodal approach including ultra-high field in vivo ^1^H MRS. We analyzed longitudinally the effect of rifaximin alone or in combination with the probiotic Vivomixx on the brain metabolic profile in the hippocampus and cerebellum of bile duct ligated (BDL) rats, an established model of type C HE. Overall, while rifaximin alone appeared to induce no significant effect on the neurometabolic profile of BDL rats, its association with the probiotic resulted in more attenuated neurometabolic alterations in BDL rats followed longitudinally (i.e. a smaller increase in Gln and milder decrease in Glu and Cr levels). Given that both rifaximin and some probiotics are used in the treatment of HE, the implications of these findings may be clinically relevant.

## Introduction

Type C hepatic encephalopathy (HE) is a neuropsychiatric disease caused by hepatic dysfunction secondary to chronic liver disease (CLD). Type C HE is characterized by neurometabolic changes, in particular an increase in brain glutamine (Gln) due to cerebral ammonium (NH_4_^+^) removal by glutamine synthetase, causing osmotic stress and secondary disruption in astrocyte metabolism^1^. It appears that this is only partially compensated by the gradual decrease of other brain metabolites (myo-inositol, taurine, total choline and creatine) from the cell^2–5^. While ammonium plays an indisputable and central role in the pathogenesis of HE, additional factors are known to contribute such as gut flora modifications, systemic inflammation, neuroinflammation and oxidative stress, all of which may precipitate the onset of HE^6,7^.

Management of type C HE remains an important challenge because treatment options are limited. Therapeutic approaches for treating HE have focused on reducing plasma ammonium levels either by decreasing its production or promoting its removal. In patients, such treatments imply the usage of non-absorbable disaccharides such as lactulose, which is accepted to improve cognitive function in patients with minimal HE (mHE), the covert premise of type C HE^8^. While lactulose is effective, it comes with side-effects including bloating, diarrhea and nausea, which limit its use^9^. Other therapeutic options include the non-absorbable antibiotic rifaximin or the administration of probiotics. These therapies act on the modulation of gut flora, in line with the current evidence that gut flora plays an important role in type C HE.

**Rifaximin** is a broad-spectrum non–absorbable antibiotic, commonly used to treat HE. The division of colonic bacteria responsible for urea deamination is thereby inhibited, reducing the production of gut ammonium and preventing absorption through the gastrointestinal tract^10^. However, different mechanisms of action have been proposed underlying the effects of rifaximin. Rifaximin may affect gut microbiota and modulate inflammatory signals in one of three ways: either by promoting anti-inflammatory cytokines such as IL-10^11^, by reducing pro-inflammatory mediators (IL-6, TNF-α) or by reducing systemic endotoxin levels^12^, none of these clearly contributing to lowering ammonia absorption from the gastrointestinal tract. Nonetheless, rifaximin has been shown to improve cognition in patients with mHE and to prevent hospitalizations and the recurrence of overt HE episodes if combined with lactulose^9,13^. However, the combined administration of rifaximin with lactulose in many of these studies is an important limitation to assess the efficacy of the rifaximin alone^14^.

Animal models of type C HE are necessary to improve the understanding of the effects of these treatments. Very few studies evaluating the effect of rifaximin and/or probiotics have been performed in animal models of type C HE^15,16^. One recent study suggested that rifaximin (50 mg/kg/day) did not reduce inflammation (TNF-α levels) and fibrosis in bile duct ligated rats (BDL)^17^, but there is a lack of knowledge about how rifaximin administration impacts changes in brain metabolites, which are frequently present in HE. Therefore, further studies are needed to characterize the potential role and mechanisms of action of rifaximin in the setting of type C HE^18^.

**Probiotics** modulate the gut bacterial flora by altering the microbial population, thereby decreasing the production of gut derived bacterial toxins^19^ and promoting non-ammoniogenic bacteria. A probiotic formulation containing a mixture of eight strains (Vivomixx in EU, Visbiome in USA) has been recently shown to improve cognitive function and inflammatory response in patients with cirrhosis^20^. This probiotic formulation has also been associated with significant improvement in minimal HE symptoms in humans, decreasing hospitalization rates and preventing encephalopathy episodes in patients with cirrhosis^21^. While the effect of probiotics was often similar to lactulose, they were better tolerated clinically^22,23^. Further, it is important to emphasize that there is significant variability among published studies in the strains of probiotics, daily dose or the duration of the administration of the probiotics, all of which potentially influencing treatment efficacy.

To date, there are very few reports in animal models of type C HE using probiotics. D’Mello et al. found that probiotics (formulation sold as VSL#3 until 2016, but now exclusively available under the brands Vivomixx and Visbiome) improved inflammation-associated sickness behavior in a mouse model of liver inflammation^24^, while Lactobacillus rhamnosus GG reduced liver fibrosis and hepatic gene expression of IL-6 in BDL rats^25^. Our group has recently shown that the administration of the probiotic Vivomixx had a beneficial effect on the development of HE in BDL rats. Both the hippocampal neurometabolic profile and the performance in behavioral tests were improved in BDL + probiotic rats compared to the BDL group at week 8 post-BDL^16^.

The effects of both the antibiotic rifaximin and probiotics have been evaluated through neurological testing in humans. They are currently used to reduce the symptoms of HE in some clinical settings, but longitudinal studies assessing their effects on brain metabolism are lacking, and the molecular mechanisms underpinning their effects are not fully understood^26^. Therefore, we aimed to use the advantages of highly resolved ^1^H MRS to analyze longitudinally, in an established model of type C HE, the effect of the antibiotic rifaximin alone or in combination with the probiotic Vivomixx on the neurometabolic profile of BDL rats. We focused on the hippocampus and cerebellum, as they are key regions involved in HE^27,28^. We hypothesized that a lesser increase in brain glutamine would be observed in the treated animals compared to the non-treated ones, followed by a milder decrease in osmolytes and neurotransmitters. We also expected that probiotics and rifaximin would act synergistically to induce better outcomes than rifaximin alone. The present study evaluates for the first time the effect the antibiotic rifaximin and its use associated with a specific probiotic formulation on the neurometabolic changes both in vivo and longitudinally in a rat model of type C HE.

## Material and methods

### Study design

Adult Wistar male rats (150–175 g, Charles River Laboratories, L’Arbresle, France) underwent bile duct ligation (BDL), a model of chronic liver disease-induced type C HE^29^. Two groups of BDL rats were used. The first group was treated with rifaximin at human pharmacological doses (‘rifaximin’, n = 12, 15.7 mg/kg/day). It was administered orally twice daily in the morning (8 am) and in the evening (6 pm) starting 2 weeks after BDL surgery (‘week 2’). The second group was treated with the same dose of rifaximin combined with the multi-strain probiotics mixture Vivomixx (n = 9, ‘rifaximin + probiotics’, Vivomixx in EU, Visbiome in USA, 60 billion bacteria/kg of rat). Vivomixx contains 8 lyophilized, highly viable bacterial strains: 4 lactobacilli (*Lactobacillus acidophilus* DSM24735, *L. plantarum* DSM24730, *L. paracasei* DSM24733, *L. bulgaricus* DSM24734), 3 bifidobacteria (*Bifidobacterium infantis* DSM24737, *B. longum* DSM24736, *B. breve* DSM24732) and *Streptococcus thermophilus* DSM24731. Probiotics administration started 2 weeks before BDL surgery until the end of the study. In this group, rifaximin was administrated at 8 am and 3 pm, and Vivomixx daily at 7 pm.

Starting from week 0 (before BDL surgery) and every 2 weeks thereafter, all rats underwent ^1^H MRS scan, blood sampling (sublingually), and feces collection for microbiology studies. Behavioral tests (open field test) were performed to evaluate motor activity as previously described^4^. During the MR experiments and blood sampling (in the sublingual vein), the animals were kept under 1.5–2% isoflurane anesthesia in a mixture of 50% air and 50% oxygen, with respiratory rate maintained at 60–70 breaths/min and body temperature at 37.5–38.5 °C. The study design is summarized in Table 1.

**Table 1:** Longitudinal study design.

	Week-2	Week 0 BDL/sham surgery	Week 2	Week 4	Week 6	Week 8
	Stool collection Start of probiotic treatment	Blood sampling Stool collection MRS scan	Blood sampling Stool collection Start of rifaximin treatment	Blood sampling Stool collection MRS scan Open field	Blood sampling Stool collection MRS scan Open field	Blood sampling Stool collection MRS scan Open field Sacrifice and organ collection
n_non-treated_		H:29/C:15/B:9	H:10/C:15	H:26/C:15/B:9/O-F:26	H:22/C:14/O-F:22	H:22/C:9/B:4/O-F:5
n_probiotics+rifaximin_		H:9/C:9/B:6	H:9/C:9	H:9/C:9 /B:6/O-F:9	H:9/ C:9/O-F:9	H:8/C:8/B:5/O-F:7
n_rifaximin_		H:12/C:12/B:10	H:12/C:12	H:12/C:12/B:10/O-F:11	H:11/C:11/O-F:11	H:8/C:8/B:8/O-F:8

The number of rats (n) measured in every group are indicated for each week and each type of measures: ‘H’ stands for MRS scan in the hippocampus and ‘C’ for MRS scan in the cerebellum, ‘B’ for the rats whose bifidobacteria in the feces were analysed, and ‘O-F’ for the rats who undergone open field test. Of note, for blood sampling we used the same number of rats as for the MRS scan (data not included in the table for readability).

Each animal served as its own control for all measurements. The treated groups were compared to one of our previously published groups of BDL non-treated rats (n = 29) and sham-operated rats (n = 18)^4^ for comparison of open field test and ^1^H MRS measurements in the hippocampus region. In addition, another group of our BDL non-treated rats (n = 15)^30^ was used for comparison of ^1^H MRS measurements in the cerebellum region. All animal experiments were conducted according to federal and local ethical guidelines, and the protocols were approved by the local Committee on Animal Experimentation for the Canton de Vaud (Switzerland). The present study is reported in accordance with ARRIVE guidelines.

### Biological and clinical characteristics of the BDL rats

Plasma measurements of ammonium and total bilirubin were performed on a COBAS8000 analyzer (Roche, Switzerland). Locomotor activity was assessed in the open field test as described previously^4^ at week 4, 6 and 8, before MRS scans (see supplementary material). The rationale was that locomotor activity would act as a surrogate for the fine motor deficits typical of type C HE.

### In vivo ^1^H magnetic resonance spectroscopy (^1^H MRS)

In vivo ^1^H MRS was performed on a 9.4T MRI system (Varian/Magnex Scientific, Oxford, UK) interfaced to a Varian Direct Drive console (Palo Alto, CA, USA) as previously described^4,31^. Brain metabolites longitudinal evolution was studied in the hippocampus in a voxel of interest (VOI) of 2 × 2.8 × 2 mm^3^, implicated in cognitive deficits observed in type C HE^28^ and in cerebellum in a VOI of 2.5 × 2.5 × 2.5 mm^3^, for its important role in motor control^27^ which is affected in type C HE^32^, using the SPECIAL^4,31^ sequence (TE = 2.8 ms, TR = 4000 ms, 160 averages). Metabolite concentrations were estimated using LCModel combined with an in vitro metabolite basis set of metabolites and the spectrum of macromolecules measured in vivo^33^. Unsuppressed water signal from the same VOI was used as internal reference for absolute metabolites quantification. The ultra-high magnetic field and ultra-short TE allow to detect and quantify in vivo 18 metabolites involved in osmoregulation, neurotransmission, energy or antioxidant metabolism: alanine (Ala), ascorbate (Asc), aspartate (Asp), glycerophosphocholine (GPC), phosphocholine (PCho), creatine (Cr), phosphocreatine (PCr), γ-aminobutyric acid (GABA), glucose (Glc), glutamine (Gln), glutamate (Glu), glutathione (GSH), myo-inositol (Ins), lactate (Lac), N-acetylaspartate (NAA), N-acetylaspartylglutamate (NAAG), phosphoethanolamine (PE), and taurine (Tau). Cramer-Rao lower bounds (CRLB) calculated from the LCModel were used as a reliability measure for the metabolite concentration estimate. Only metabolites with CRLB lower than 30% were included in the results.

### Bifidobacteria measurements

Feces were collected at week 0, 4 and 8 to measure bifidobacteria concentration. Measurements were performed as described previously by Mastromarino et al.^34^.

### Statistical analysis

All results are presented as mean ± SD and % of increase/decrease compared to week 0. One-way ANOVA (Prism 5.03, Graphpad, La Jolla CA USA) with respect to each metabolite in the neurochemical profile followed by Bonferroni’s multi-comparisons post-test was used within a single group.

Two-way ANOVA (Prism 5.03, Graphpad, La Jolla CA USA) followed by the Bonferroni's multi comparisons post-test was used to assess significance (*p* < 0.05) in bifidobacteria, distance moved, brain and plasma metabolite's changes between the groups ('treatment' factor). As variations in metabolite concentrations between groups may appear as early as week 0, our analysis is based on the results of the two-way ANOVA on metabolites changes both in absolute value and relative to week 0. All tests were 2-tailed. In addition, for metabolites with a significant difference between groups ('treatment' factor) both in absolute concentration and relative to week 0, additional two-way ANOVA tests were performed by comparing the ‘non-treated’ group and each of these two groups individually. This was done to eliminate the potential influence of the differences between the ‘rifaximin’ group and the ‘rifaximin + probiotics’ group.

Pearson correlation analysis was performed on longitudinally acquired data to test for correlations between brain metabolites and plasma values.

## Results

**Figure 1:**
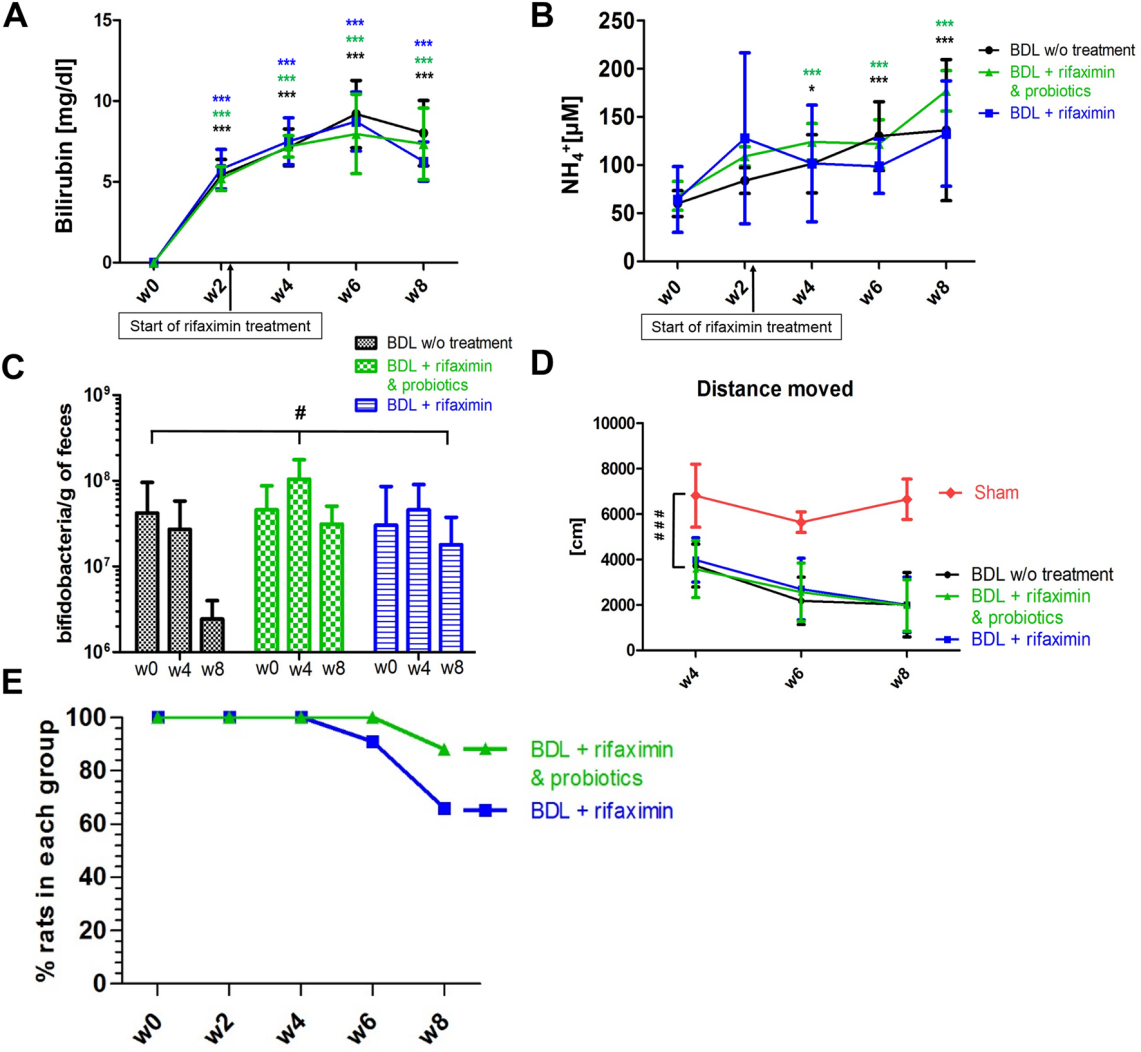
Longitudinal changes in plasma total bilirubin (**A**) and NH_4_^+^ (**B**) in the three groups. In (**A**) and (**B**), statistical significance intra-group (labelled with ‘*’) is shown. (**C**) Bifidobacteria concentration in feces in the three BDL groups. (**D**) Distance traveled by the three groups of BDL and sham-operated rats. In (**C**) and (**D**), the overall statistical significance between-groups (‘treatment’ factor, labelled with ‘#’) is shown. (**E**) Percentage of BDL rats at each week in the ‘rifaximin + probiotics’ group and in the ‘rifaximin’ group. All results are presented as mean ± SD. One-way ANOVA was used to assess the significance intra-group (**A**, **B**) and two-way ANOVA to assess significance between-groups (‘treatment’ factor; **C**, **D**). Statistical significance: *p < 0.05; **p < 0.01; ***p < 0.001.

First, the biological and clinical features of the BDL rats were characterized using plasma bilirubin levels. Bilirubin levels were increased in the three groups of rats (Fig. 1A), without significant difference between groups. All rats displayed a similar ammonium increase regardless of the group (Fig. 1B). During the open field test, the distance traveled decreased from week 4 to week 8 in all groups of BDL rats and was significantly shorter than the sham-operated animals. No significant differences were observed between the three BDL groups throughout of the study (Fig. 1D).

There were significant differences in the longitudinal evolution of the amount of bifidobacteria in feces between the three groups (two-way ANOVA, ‘treatment’ factor, *p* = 0.03, Fig. 1C). The difference was particularly pronounced at week 8 where the amount of bifidobacteria in the ‘rifaximin + probiotics’ group was on average twelve times higher compared to the ‘non-treated’ group (one-way ANOVA, *p* = 0.0106).

We further noted that the general condition of the rats in the ‘rifaximin + probiotics’ group was overall better than in the ‘rifaximin’ group, as reflected by the fact that only one rat reached the humane endpoint (euthanasia) in this group before the 8 weeks of the study compared to four rats in the ‘rifaximin’ group (Fig. 1E). Of note, the ‘non-treated’ BDL rats come from a previously published study^4^ where no such assessment was performed since some of the rats were sacrificed before reaching the humane endpoint for additional measures.

**Figure 2:**
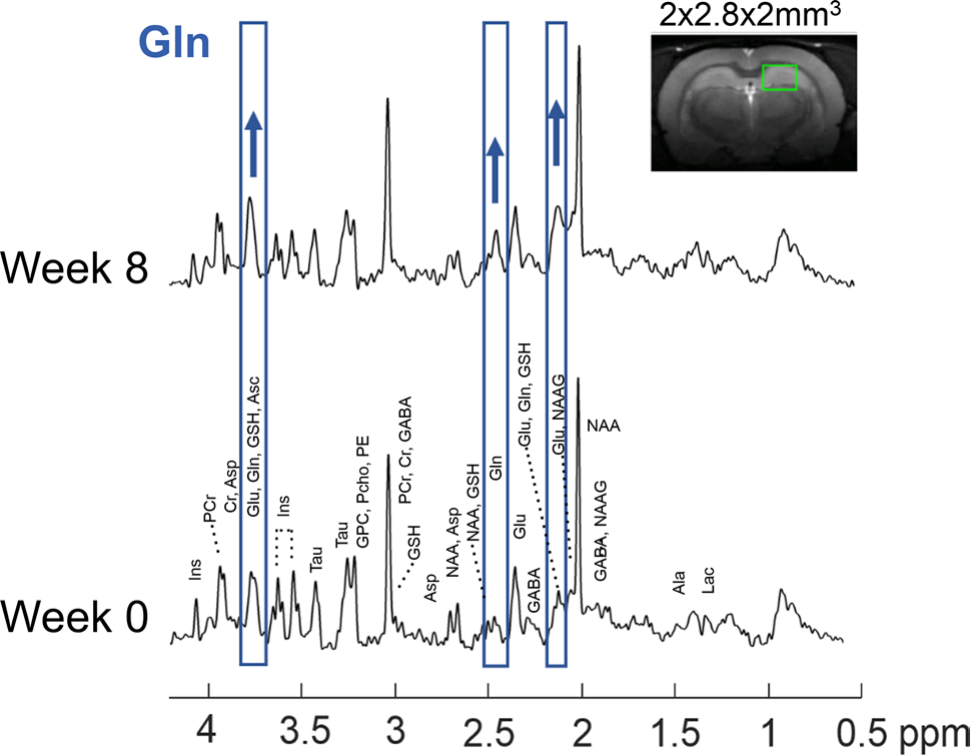
Representative ^1^H MRS spectra in the hippocampus of a ‘BDL + rifaximin & probiotics’ rat at week 0 and week 8 after BDL. Part of the spectra corresponding to Gln are shown in blue, which visibly increased from week 0 to week 8. The green rectangle shows the region considered in the hippocampus on the T_2_ weighted axial image of the rat brain.

To assess the neurometabolic consequences of BDL we performed in vivo ^1^H magnetic resonance spectroscopy (^1^H MRS) at 9.4 Tesla. The high quality of the spectra throughout the study allowed the separation of Gln from Glu and thus quantification of 16 brain metabolites (Fig. 2). All the groups exhibited a significant increase of brain Gln due to ammonia detoxification, and a subsequent reduction of other brain osmolytes (Ins, Tau, tCho), as well as antioxidants and neurotransmitters.

**Figure 3:**
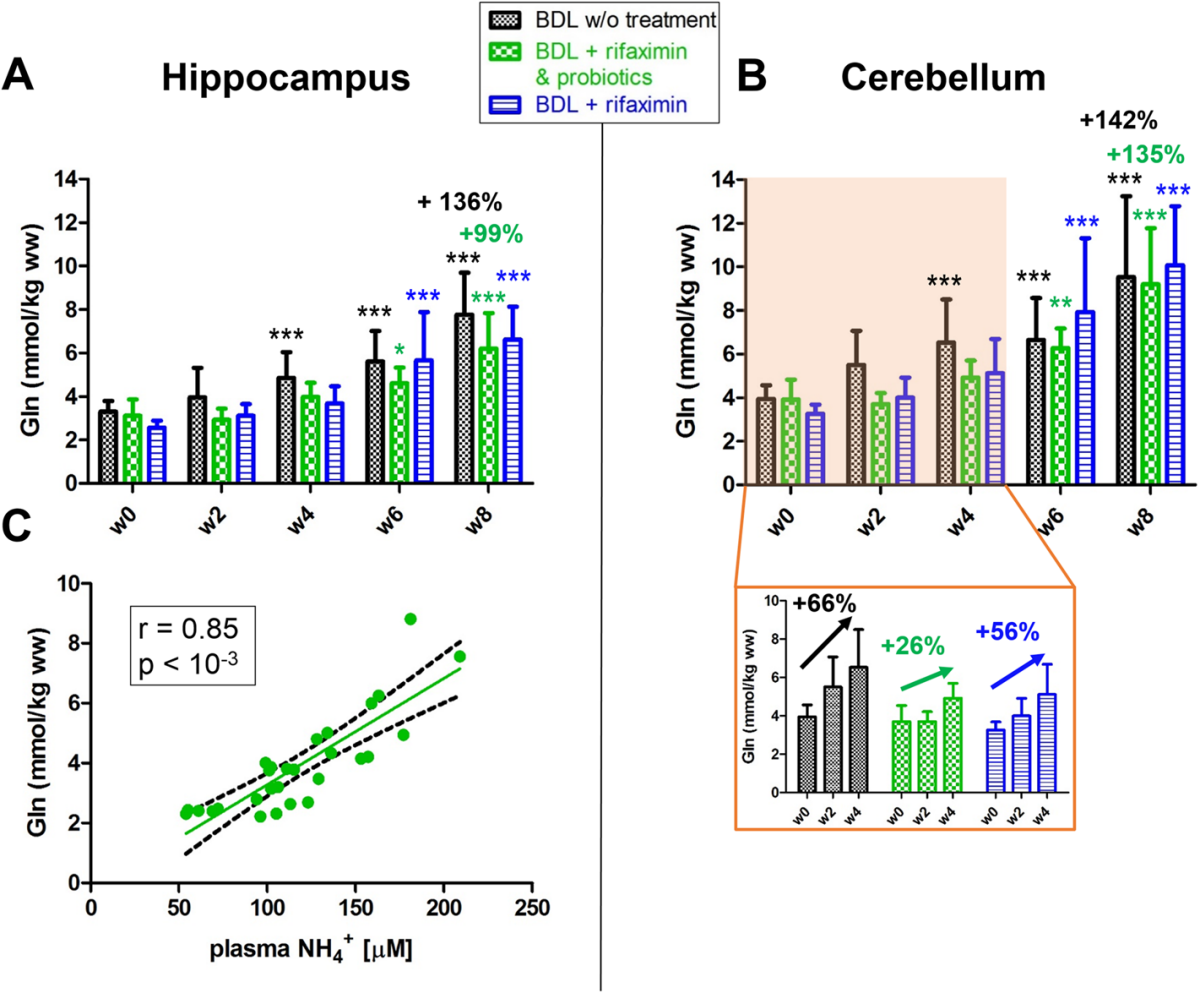
Brain Gln in the hippocampus (**A**) and in the cerebellum (**B**) from week 0 to week 8 in the three groups. Statistical significance intra-group reported: one-way ANOVA (*p < 0.05; **p < 0.01; ***p < 0.001). The relative increases shown are relative to week 0. (**C**) Correlation between brain Gln in the hippocampus and plasma ammonium in rats treated with the combination of rifaximin and probiotics.

Brain **Gln** significantly increased in all three groups both in the hippocampus and the cerebellum (Fig. 3). However, ^1^H MRS revealed significant differences in longitudinal Gln changes between the ‘rifaximin + probiotics’, the ‘rifaximin’ and the ‘non-treated’ rats: Gln increase was significantly lower in the ‘rifaximin + probiotics’ group at week 2, 4, 6, and 8 in the hippocampus compared to the ‘non-treated’ group, both in absolute value and relative to week 0 (+ 99% in the ‘rifaximin + probiotics’ group vs + 136% in the ‘non treated’ group at week 8, Fig. 3A and Table 2). Similarly, in the cerebellum region, the ‘rifaximin + probiotics’ rats showed a lower rise in brain Gln at early stage of HE compared to ‘non-treated’ rats (+ 26% in the ‘rifaximin + probiotics’ group vs + 66% in the ‘non treated’ group at week 4, Fig. 3B) with the differences observed between groups being significant both in absolute value and relative to week 0 (Table 2). However, no significant differences were observed between ‘rifaximin’ rats and the ‘non-treated’ rats, even though the % increase in Gln was higher in the ‘rifaximin’ group.

**Table 2:** Summary of statistical significance of the two-way ANOVA for ‘treatment’ factor (i.e. ‘rifaximin’, ‘rifaximin + probiotics’, ‘non-treated’) for each metabolites whose concentrations significantly varied over time, both in absolute concentrations and relative to week 0 in hippocampus and cerebellum.

Two way ANOVA—significance for ‘treatment’ factor	Hippocampus		Cerebellum	
	Absolute concentrations	Relative to week 0	Absolute concentrations	Relative to week 0
Gln	***	**	*	*
Ins	***	ns	***	ns
tCho	*	ns	ns	ns
Tau	ns	ns	ns	ns
Cr	ns	ns	**	*
tCr	*	ns	ns	ns
Lac	ns	ns	***	ns
Glu	***	ns	*	**
Asc	***	ns	ns	ns

Metabolites which significantly differ between the groups both in absolute and relative values are highlighted in red. Of note, the interaction was not significant for any of the metabolites. The relative increase reported are relative to week 0. Statistical significance: *p < 0.05; **p < 0.01; ***p < 0.001.

Furthermore, brain Gln in the hippocampus correlated positively with blood ammonium in the ‘rifaximin + probiotics’ group (*r* = 0.85, *p* < 10^−3^, Fig. 3C), while the correlation did not reach significance in the ‘rifaximin’ group (*r* = 0.27, *p* = 0.056). The correlations between cerebellar Gln and plasma ammonium were similar to those observed in the hippocampus (*r* = 0.83, *p* < 10^−3^ in the ‘rifaximin + probiotics’ group; *r* = 0.26, *p* = 0.081 in the ‘rifaximin’ group). Interestingly, in both treated groups the distance moved in the open field test correlated negatively with Gln in the cerebellum, an important region implicated in motor control (*r* =  − 0.51, *p* = 0.009 in the ‘rifaximin + probiotics’ group; *r* =  − 0.65, *p* = 0.0001 in the ‘rifaximin’ group).

**Figure 4:**
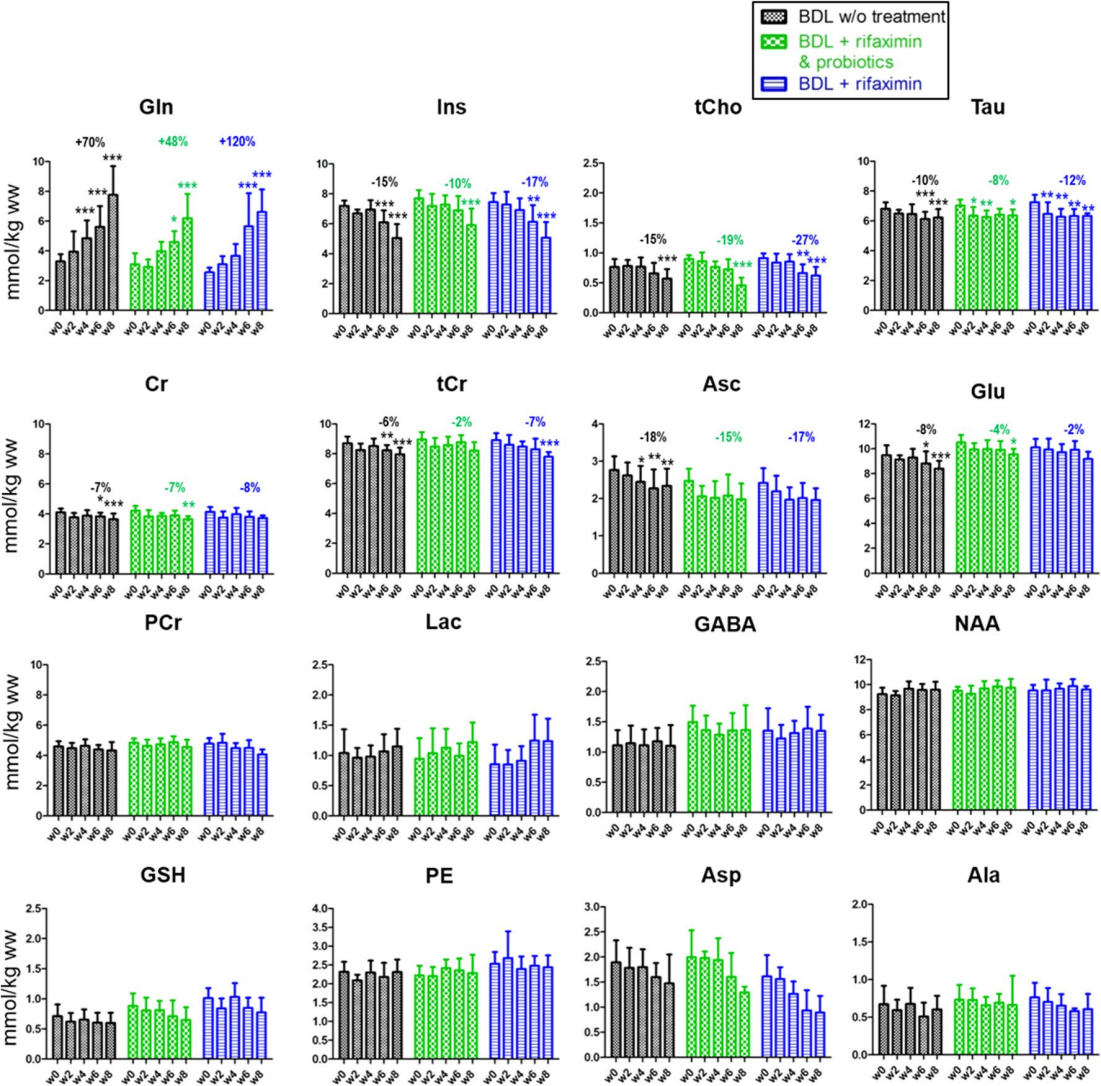
Longitudinal ^1^H MRS data of brain metabolites in the hippocampus region of the ‘rifaximin’ treated group, the ‘rifaximin + probiotics’ treated group and the group without treatment. For better readability, relative changes indicated in the graph are shown only at week 6 relative to week 0 and are indicated for metabolites whose concentrations significantly varied over time. Statistical significance: One-way ANOVA (*p < 0.05; **p < 0.01; ***p < 0.001).

The decrease of the osmolyte Ins was significant in all groups in both hippocampus and in cerebellum. It was less marked in the ‘rifaximin + probiotics’ group compared to the other groups at week 6 and at week 8, both in absolute value and relative to week 0 (− 10% in the ‘rifaximin + probiotics’ group vs − 15% in the ‘non treated’ group at week 6, Fig. 4).

**Figure 5:**
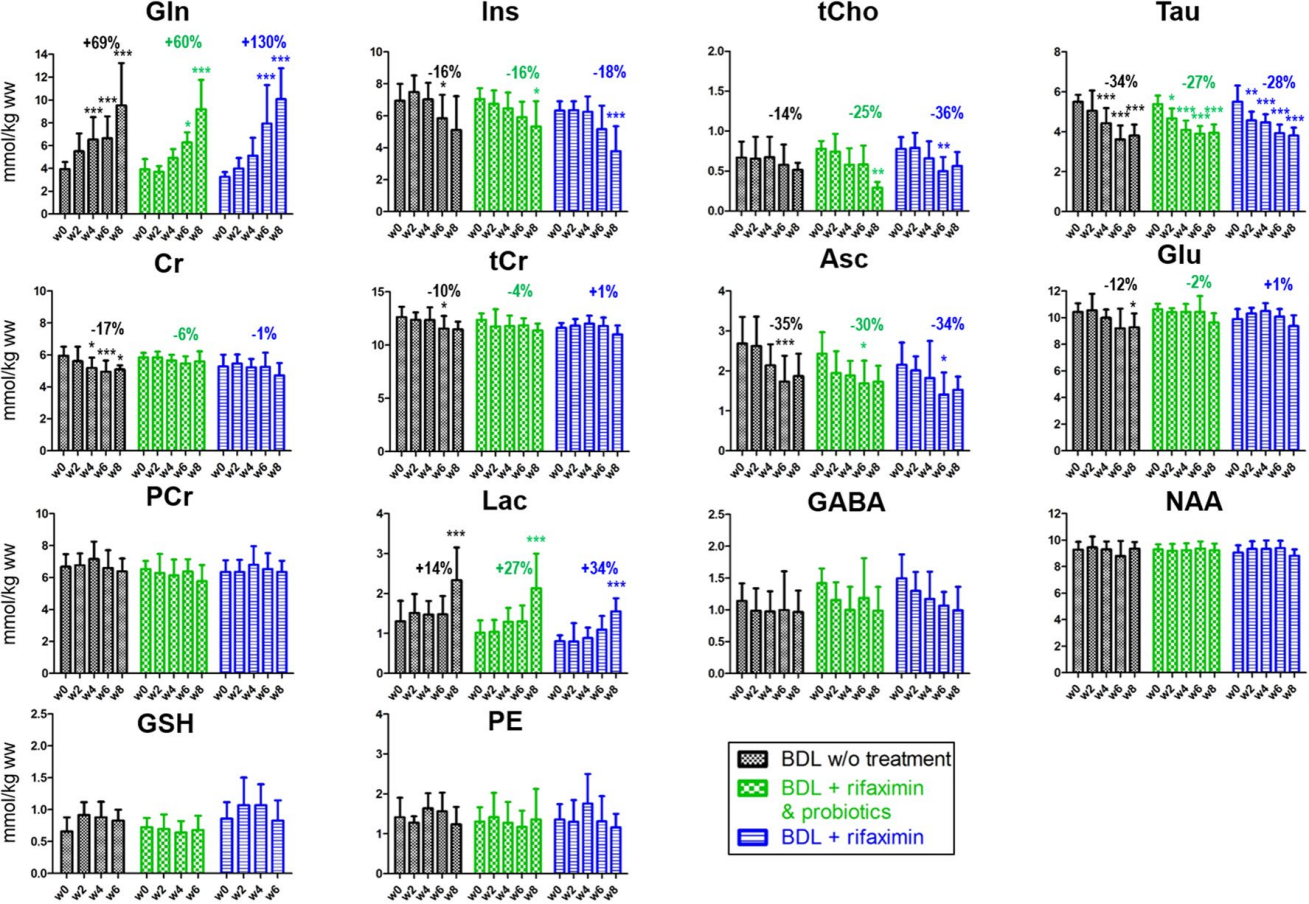
Longitudinal ^1^H MRS data of the brain metabolites in the cerebellum region of the ‘rifaximin’ treated group, the ‘rifaximin + probiotics’ treated group and the group without treatment. Relative changes indicated in the graph at week 6 are calculated relative to week 0 and are indicated for metabolites whose concentrations significantly varied over time. Statistical significance: One-way ANOVA (*p < 0.05; **p < 0.01; ***p < 0.001). Note that in the cerebellum many concentrations estimated with LCModel of Asp and Ala were excluded due to their unreliability (CRLB > 30%). As this made difficult to interpret the variations of these metabolites, their evolutions are not shown. GSH at week 8 is not shown for the same reason.

Among the other brain organic osmolytes, both Tau and tCho concentrations decreased significantly in all three groups in both the hippocampus and cerebellum, with no significant difference between groups (Figs. 4 and 5).

Cr, a metabolite known for its role in energy metabolism and reported to participate in osmoregulation and neuroprotection^35^ showed a decrease in the hippocampus in the three groups without any significant difference between groups. However, in the cerebellum, Cr concentration was stable over time in the ‘probiotics + rifaximin’ group (− 4% at week 8, Fig. 5), while its decrease was stronger in the two other groups (− 11% in the ‘rifaximin’ group vs − 15% in the ‘non-treated’ group at week 8, Fig. 5). These differences between groups were significant both in absolute and relative values (Fig. 5 and Table 2). Although not reaching significance, there was a trend toward an attenuated reduction of tCr in the ‘rifaximin + probiotics’ group compared to the ‘non-treated’ group both in the hippocampus (− 2% vs − 6% at week 6) and in cerebellum (− 4% vs − 10% at week 6).

Among the neurotransmitter-related metabolites, Glu decrease was less marked in the ‘rifaximin + probiotics’ group and in the ‘rifaximin’ groups compared to the ‘non-treated’ group in both hippocampus (− 4% in the ‘rifaximin + probiotics’ group vs − 8% in the ‘non-treated’ group at week 6, Fig. 4) and cerebellum (− 2% in the ‘rifaximin + probiotics’ group vs − 15% in the ‘non-treated’ group at week 6, Fig. 5). Interestingly, these differences between groups were significant only in the cerebellum (Table 2). Asp decreased similarly between groups without reaching significance (Fig. 4).

When considering antioxidants measurable by ^1^H MRS, the Asc decrease was slightly less marked in the two treated groups while no significant changes were observed for GSH.

Lac exhibited a slight tendency to increase its concentration in the three groups in the hippocampus, but none of these changes reached significance. In the cerebellum, however, the Lac increase was more substantial and significant at week 8 in all groups (+ 80%, + 109%, + 92% at week 8 in the ‘non-treated’, ‘rifaximin + probiotics’, ‘rifaximin’ groups respectively) but did not significantly vary between the groups (Figs. 4, 5 and Table 2). GABA, PE, NAA, NAAG nor tNAA did not change significantly during the course of the study in any of the groups or brain region.

We also measured the correlations between Gln, for its central role in the development of HE, and other neurometabolites to assess inter-metabolite dependencies in treated BDL rats. Overall, with each of the two treatments, the hippocampal Gln increase was strongly correlated with the decrease in Ins (*p* < 0.001 for both groups) and in tCho (*p* < 0.001 for both groups). We observed a negative correlation between Gln and Glu, which was more pronounced in the ‘rifaximin + probiotics’ group (*r* =  − 0.36, *p* = 0.017) compared to the ‘rifaximin’ group (*r* =  − 0.29, *p* = 0.027). In the cerebellum region, Gln also correlated negatively with Ins in both treated groups (*p* < 0.001 in both groups) and with tCho (*p* < 0.01 in both groups). Gln correlated negatively with Glu in the ‘probiotics + rifaximin’ group only (*p* < 0.01). Unlike the hippocampal region, a strong correlation was measured with Tau (*p* < 0.001 in both groups).

Furthermore, for metabolites with a significant difference between groups ('treatment' factor) both in absolute concentration and relative to week 0 (Gln, Cr, Glu, Table 2) as well as for bifidobacteria (Fig. 1C), we performed an additional two-way ANOVA test to compare only the ‘non-treated’ group and the ‘rifaximin + probiotics’ group. The differences between the groups were all significant: Gln hippocampus, *p* < 0.001; Gln cerebellum, *p* < 0.05; Glu cerebellum, *p* < 0.05; Cr cerebellum, *p* < 0.01; bifidobacteria, *p* < 0.05.

## Discussion

The present study reports for the first time the beneficial effect of combined rifaximin and the probiotic Vivomixx on the longitudinal neurometabolic changes in vivo in a rat model of type C HE. The characteristic longitudinal increase of Gln and decrease of Glu, known to characterize HE, and the recently reported decreased in Cr, were significantly less pronounced in rats treated with rifaximin and probiotics compared to the two other groups.

In each group, we observed the characteristic features of type C HE previously described using ^1^H MRS^4^ (Fig. 2). In all three groups, there was a gradual increase of brain Gln followed by a gradual decrease in Ins and other osmolytes, such as Tau and Cr. These observations are in agreement with previously published studies in BDL rats and CLD patients^3,4,36^ and confirm the already well-known partial compensatory effect to maintain osmotic balance in the presence of Gln accumulation.

The measured plasma bilirubin values as well as the lack of significant changes in treated animals are in agreement with recently published studies on BDL rats treated with probiotics^16,25^. We observed a significant increase in plasma ammonium in all groups to a level similar to that reported in other studies using BDL rats^4,37^, with a high intra-group variability. No significant differences were observed between groups, despite the administration of rifaximin combined with probiotics or rifaximin alone, whose purported role is to reduce the production of gut ammonium. Of note, the lack of significance in plasma NH_4_^+^ measurements between groups is in agreement with a recently published study on BDL rats^16^. However, the effect of rifaximin on plasma ammonium levels is still disputed, and some studies reported that its use had no significant effect on plasma ammonium levels either in BDL rats^15^ or in humans^11^. In contrast, some studies reported a reduction in blood ammonium after treatment^38,39^, but it is now generally considered that despite the irrefutable and central role of ammonium in the pathogenesis of HE, its plasma levels display inter-individual variability and are not always useful to predict the severity of HE or to assess the response to therapy, especially in type C HE^40,41^. Given these aforementioned elements and the challenges of measuring ammonium, additional methods must be combined in a multi-modal approach to accurately assess the effect of different therapeutic options in type C HE, as put forth in the present study.

Gln increase was significantly less pronounced in the ‘rifaximin + probiotics’ group at weeks 4, 6 and 8, compared to the two other groups, in agreement with a recent study using the probiotic Vivomixx alone on the same animal model^16^. Therefore, the significantly lower decrease of brain Glu in the ‘rifaximin + probiotics’ group suggests that the reduction in brain Glu is mainly the result of ammonium detoxification through glutamine synthetase, which catalyzes the formation of Gln from ammonium and glutamate in astrocytes^42^. This might also explain the positive correlation between brain Gln in hippocampus and plasma ammonium in the ‘rifaximin + probiotics’ group. The significant correlation between Gln and Glu (in all of the three groups) is consistent with this hypothesis. In addition, the correlations observed between hippocampal Gln, Ins, Tau and plasma NH_4_^+^ are in agreement with the previously published results in non-treated BDL rats^4^.

While Glu showed a significantly less pronounced decrease in the ‘rifaximin + probiotics’ group, Asp, a selective NMDA receptor agonist, showed a similar trend of decreasing concentration in all groups, although not significant (Fig. 4). Even if the role of Asp as an excitatory neurotransmitter is still debated, its decrease might be linked to alteration in the neurotransmission system, which is known to play an essential role in the pathophysiology of HE even though the precise mechanisms are not yet fully identified^43^. In addition, no significant changes in the inhibitory neurotransmitter GABA concentrations were observed, consistent with our previous studies in non-treated BDL rats^4^.

An attenuated decrease of Cr, tCr and Ins was measured in the ‘rifaximin + probiotics’ group, consistent with previous reports^16^. These smaller variations of Cr and tCr, metabolites also involved in energy metabolism, suggest a lower osmotic imbalance and possibly improvement of the energy metabolism in the ‘rifaximin + probiotics’ group.

Regarding microbiota, rifaximin alone did not significantly alter bifidobacteria concentration in feces. This is in keeping with two previously reported observations: first, that no significant change in microbial composition after rifaximin administration in humans was observed^44^, and second, that rifaximin may exert a beneficial modulation of the gut microbiota metabolic profile rather than a significant rearrangement of the intestinal microbial community^45^. The probiotics used in our study resulted in a significantly higher bifidobacteria concentration at week 4 and week 8 in the intestines of rats treated with rifaximin combined with Vivomixx compared to the two other groups. These modifications may have decreased the production and absorption of ammonium, as reported in various studies in humans^23,46^ and might explain the lower brain Gln measured in this group. These results are consistent with a previous preliminary study showing an increase of bifidobacteria in gut microbiota in BDL rats treated with the probiotic Vivomixx only^16^.

The present study has several limitations. First, stool bifidobacteria concentrations were assessed at only 3 time points (weeks 0, 4 and 8) using a limited number of samples. Moreover, alterations in gut microbiota composition and function in CLD contribute to the systemic inflammation and accumulation of gut-derived toxins^47^, something for which we could not control in the present study. As both rifaximin and probiotics influence systemic inflammation^18^, the longitudinal assessment of inflammatory markers in the blood (TNF-α, CRP, WBC counts) and markers of neuroinflammation remains to be performed.

It is important to emphasize that different factors influenced our study design: first, various studies suggested that probiotics improve antibiotic therapy as they possess immunomodulatory properties, promote the recovery of commensal microbiota and increase treatment tolerability^48^. To date, the evidence is unclear as to the best time to administer probiotics. For now, patients with type C HE are advised to take probiotics before, at the same time as, or even after taking antibiotics. Second, one purpose of the present study was to compare two similar groups. Therefore, modulation of the gut flora started 2 weeks before BDL surgery in the ‘rifaximin + probiotics’ group and rifaximin was administered at the same dosage per day in both treated groups (‘rifaximin’ and ‘rifaximin + probiotics’). In the ‘rifaximin + probiotics’ group, drug administration was timed carefully (alternating times for rifaximin and probiotics) in order to minimize any direct interaction in the gastrointestinal tract between the two treatments. These strategies used in our experimental design may have affected the magnitude of the results.

Overall, while rifaximin alone at this specific dose appeared to induce no significant effect on the neurometabolic profile of treated—compared to non-treated BDL rats, its association with probiotics resulted in more attenuated neurometabolic alterations in BDL rats followed longitudinally. Our results on rifaximin alone are consistent with a recent study that explored the effects of rifaximin in BDL rats, where the authors concluded that rifaximin at this dose was not efficacious in the treatment of HE^49^. We thus hypothesize that the positive effects observed are mainly due to indirect effects of this specific probiotic formulation, namely modulation of the intestinal barrier, anti-inflammatory properties, and decreased production of bacterial toxins^19^. Our results are in keeping with our recent study showing a beneficial effect of the probiotic Vivomixx alone in BDL rats^16^.

## Conclusion

We conclude that in BDL rats rifaximin treatment combined with the probiotic Vivomixx can be associated with positive effects on brain Gln, Glu and Cr levels in a model of type C HE. Given that both rifaximin and some probiotics are used in the treatment of HE, the clinical implications of these findings may be far-reaching.

## Supplementary Information

Supplementary Information 1.

## References

[1] Brusilow SW, Koehler RC, Traystman RJ, Cooper AJL (2010). Astrocyte glutamine synthetase: Importance in hyperammonemic syndromes and potential target for therapy. Neurotherapeutics.

[2] Braissant O, McLin VA, Cudalbu C (2013). Ammonia toxicity to the brain. J. Inherit. Metab. Dis..

[3] Häussinger D (2006). Low grade cerebral edema and the pathogenesis of hepatic encephalopathy in cirrhosis. Hepatology.

[4] Braissant O, Rackayová V, Pierzchala K, Grosse J, McLin VA, Cudalbu C (2019). Longitudinal neurometabolic changes in the hippocampus of a rat model of chronic hepatic encephalopathy. J. Hepatol..

[5] Lanz B, Rackayova V, Braissant O, Cudalbu C (2017). MRS studies of neuroenergetics and glutamate/glutamine exchange in rats: Extensions to hyperammonemic models. Anal. Biochem..

[6] Coltart I, Tranah TH, Shawcross DL (2013). Inflammation and hepatic encephalopathy. Arch. Biochem. Biophys..

[7] Azhari H, Swain MG (2018). Role of peripheral inflammation in hepatic encephalopathy. J. Clin. Exp. Hepatol..

[8] Prasad S, Dhiman RK, Duseja A, Chawla YK, Sharma A, Agarwal R (2007). Lactulose improves cognitive functions and health-related quality of life in patients with cirrhosis who have minimal hepatic encephalopathy. Hepatology.

[9] Gitlin, N. Treatment of hepatic encephalopathy with rifaximin: More to think about. *Hepatology* **53** (3), 1059; author reply 1059–1060. 10.1002/hep.24112 (2011). 10.1002/hep.24112 21294145

[10] Compendium. (Accessed 8 January 2020); https://compendium.ch/fr/product/1292134-xifaxan-filmtabl-550-mg.

[11] Bajaj JS, Heuman DM, Wade JB (2011). Rifaximin improves driving simulator performance in a randomized trial of patients with minimal hepatic encephalopathy. Gastroenterology.

[12] Kalambokis GN, Tsianos EV (2012). Rifaximin reduces endotoxemia and improves liver function and disease severity in patients with decompensated cirrhosis. Hepatology.

[13] Bass NM, Mullen KD, Sanyal A (2010). Rifaximin treatment in hepatic encephalopathy. N. Engl. J. Med..

[14] Hadjihambi A, Arias N, Sheikh M, Jalan R (2017). Hepatic encephalopathy: A critical current review. Hepatol. Int..

[15] Thabut D, Mouri S, El Mourabit H (2015). Sodium benzoate and rifaximin are able to restore blood-brain barrier integrity in he cirrhotic rats. Intensive Care Med. Exp..

[16] Rackayova V, Flatt E, Braissant O (2021). Probiotics improve the neurometabolic profile of rats with chronic cholestatic liver disease. Sci. Rep..

[17] Shin SK, Kwon OS, Lee JJ (2017). Effect of rifaximin on hepatic fibrosis in bile duct-ligated rat model. Korean J. Gastroenterol..

[18] Bajaj JS (2016). Review article: Potential mechanisms of action of rifaximin in the management of hepatic encephalopathy and other complications of cirrhosis. Aliment Pharmacol. Ther..

[19] Rowland I, Capurso L, Collins K (2010). Current level of consensus on probiotic science–report of an expert meeting–London, 23 November 2009. Gut Microb..

[20] Román E, Nieto JC, Gely C (2019). Effect of a multistrain probiotic on cognitive function and risk of falls in patients with cirrhosis: A randomized trial. Hepatol. Commun..

[21] Lunia MK, Sharma BC, Sharma P, Sachdeva S, Srivastava S (2014). Probiotics prevent hepatic encephalopathy in patients with cirrhosis: A randomized controlled trial. Clin. Gastroenterol. Hepatol..

[22] Saab S, Suraweera D, Au J, Saab EG, Alper TS, Tong MJ (2016). Probiotics are helpful in hepatic encephalopathy: A meta-analysis of randomized trials. Liver Int..

[23] Pratap Mouli V, Benjamin J, Bhushan Singh M (2015). Effect of probiotic VSL#3 in the treatment of minimal hepatic encephalopathy: A non-inferiority randomized controlled trial. Hepatol. Res..

[24] D’Mello C, Ronaghan N, Zaheer R (2015). Probiotics improve inflammation-associated sickness behavior by altering communication between the peripheral immune system and the brain. J. Neurosci..

[25] Hammes TO, Leke R, Escobar TDC (2017). Lactobacillus rhamnosusGG reduces hepatic fibrosis in a model of chronic liver disease in rats. Nutr. Hosp..

[26] Swaminathan M, Ellul MA, Cross TJ (2018). Hepatic encephalopathy: Current challenges and future prospects. Hepat. Med..

[27] Manto M, Bower JM, Conforto AB (2012). Consensus paper: Roles of the cerebellum in motor control–the diversity of ideas on cerebellar involvement in movement. Cerebellum.

[28] Bahceci F, Yildirim B, Karincaoglu M, Dogan I, Sipahi B (2005). Memory impairment in patients with cirrhosis. J. Natl. Med. Assoc..

[29] Butterworth RF, Norenberg MD, Felipo V (2009). Experimental models of hepatic encephalopathy: ISHEN guidelines. Liver Int..

[30] Simicic D, Pierzchala K, Rackayová V (2019). P: 33 in vivo longitudinal 1H MRS study of hippocampal, cereberal and striatal metabolic changes in the adult brain using an animal model of chronic hepatic encephalopathy. Am. J. Gastroenterol..

[31] Rackayova V, Braissant O, Rougemont A-L, Cudalbu C, McLin VA (2020). Longitudinal osmotic and neurometabolic changes in young rats with chronic cholestatic liver disease. Sci. Rep..

[32] Kharbanda PS, Saraswat VA, Dhiman RK (2003). Minimal hepatic encephalopathy: Diagnosis by neuropsychological and neurophysiologic methods. Indian J. Gastroenterol..

[33] Cudalbu C, Behar KL, Bhattacharyya PK (2020). Contribution of macromolecules to brain 1 H MR spectra: Experts’ consensus recommendations. NMR Biomed..

[34] Mastromarino P, Capobianco D, Campagna G (2014). Correlation between lactoferrin and beneficial microbiota in breast milk and infant’s feces. Biometals.

[35] Rackayova V, Cudalbu C, Pouwels PJW, Braissant O (2017). Creatine in the central nervous system: From magnetic resonance spectroscopy to creatine deficiencies. Anal. Biochem..

[36] Córdoba J, Alonso J, Rovira A (2001). The development of low-grade cerebral edema in cirrhosis is supported by the evolution of (1)H-magnetic resonance abnormalities after liver transplantation. J. Hepatol..

[37] Aamann L, Ochoa-Sanchez R, Oliveira M (2019). Progressive resistance training prevents loss of muscle mass and strength in bile duct-ligated rats. Liver Int..

[38] Pedretti G, Calzetti C, Missale G, Fiaccadori F (1991). Rifaximin versus neomycin on hyperammoniemia in chronic portal systemic encephalopathy of cirrhotics. A double-blind, randomized trial. Ital. J. Gastroenterol..

[39] Paik YH, Lee KS, Han KH (2005). Comparison of rifaximin and lactulose for the treatment of hepatic encephalopathy: A prospective randomized study. Yonsei Med. J..

[40] Nicolao F, Efrati C, Masini A, Merli M, Attili AF, Riggio O (2003). Role of determination of partial pressure of ammonia in cirrhotic patients with and without hepatic encephalopathy. J. Hepatol..

[41] Mallet M, Weiss N, Thabut D, Rudler M (2018). Why and when to measure ammonemia in cirrhosis?. Clin. Res. Hepatol. Gastroenterol..

[42] Cooper AJ, Plum F (1987). Biochemistry and physiology of brain ammonia. Physiol. Rev..

[43] Felipo V (2013). Hepatic encephalopathy: Effects of liver failure on brain function. Nat. Rev. Neurosci..

[44] Ridlon JM, Alves JM, Hylemon PB, Bajaj JS (2013). Cirrhosis, bile acids and gut microbiota: Unraveling a complex relationship. Gut Microb..

[45] Ponziani FR, Gerardi V, Pecere S (2015). Effect of rifaximin on gut microbiota composition in advanced liver disease and its complications. World J. Gastroenterol..

[46] Sharma P, Sharma BC, Puri V, Sarin SK (2008). An open-label randomized controlled trial of lactulose and probiotics in the treatment of minimal hepatic encephalopathy. Eur. J. Gastroenterol. Hepatol..

[47] Campion D, Giovo I, Ponzo P, Saracco GM, Balzola F, Alessandria C (2019). Dietary approach and gut microbiota modulation for chronic hepatic encephalopathy in cirrhosis. World J. Hepatol..

[48] Boyanova L, Mitov I (2012). Coadministration of probiotics with antibiotics: Why, when and for how long?. Expert Rev. Anti Infect. Ther..

[49] Petrazzo, G. *Cibler le système digestif pour protéger le foie: évaluation de l’efficacité prophylactique et thérapeutique de traitements de l’encéphalopathie hépatique dans un modèle murin de cholestase hépatique par ligature de la voie biliaire* (March 2020, accessed 6 July 2020); https://papyrus.bib.umontreal.ca/xmlui/handle/1866/23673.

