# Supplementary material for: Probiotics combined with rifaximin influence the neurometabolic changes in a rat model of type C HE
Source: Sci Rep. 2021 Sep 9;11:17988. doi: 10.1038/s41598-021-97018-8 (PMC8429411; doi:10.1038/s41598-021-97018-8)
Supplement: Supplementary file 1 — Supplementary Information 1. [file 41598_2021_97018_MOESM1_ESM.docx]

**Supplementary material**

*MRS/MRI procedures and biochemical measurements*

During all the MRS/MRI procedures and biochemical measurements, animals were anesthetized using 1.5–2 % of isoflurane in 50 % air / 50 % oxygen. Respiration rate was maintained at 60–70 breaths/min and body temperature at 37.5–38.5 °C. Animals had unrestricted access to standard rat food and water for the duration of study. All procedures were approved by the Committee on Animal Experimentation for the canton of Vaud, Switzerland (authorization 2812).

*Behavioral tests: open field*

In order to evaluate motor activity in the different groups, the open field test was used. This test analyzes the rodent's exploratory behavior in a confined space and is mainly used to measure its motor functions. Open field test was performed at week 4, 6 and 8 after BDL surgery. The rat was placed in an open circular arena divided into three concentric virtual part of different light intensities. Its behavior was recorded for 10 minutes. Different parameters were evaluated with the video tracking system (Noldus Ethovision software 11.5) such as distance moved (cm) and velocity. These parameters were calculated for the 10 minutes of the test.

**
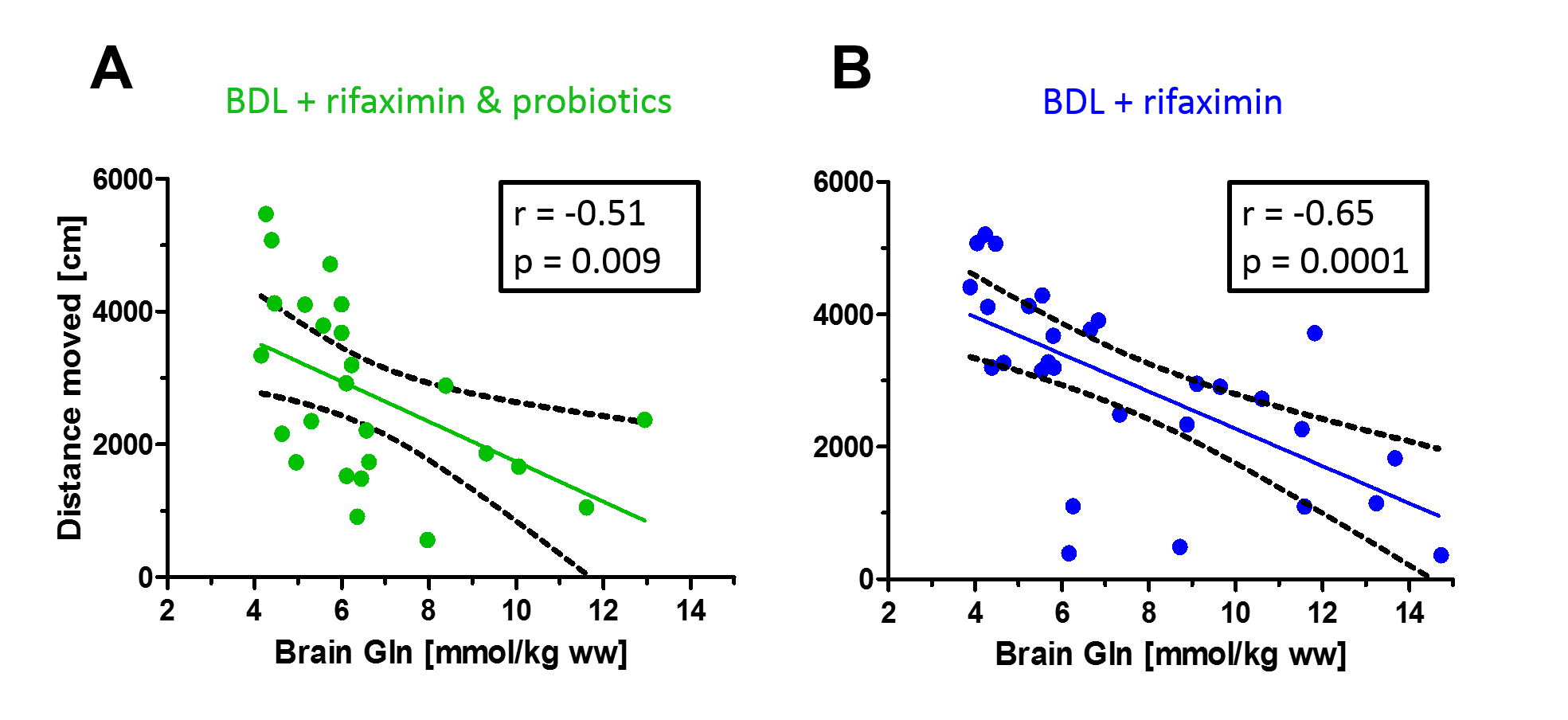
**

**Supplementary Figure 1** Correlation between brain Gln in the cerebellum and the distance moved in the open field test in BDL rats treated with the combination of rifaximin and probiotics (A) and in BDL rats treated with rifaximin only (B).
